# Art therapy with an African American female combat veteran experiencing effects of mild traumatic brain injury and post-traumatic stress

**DOI:** 10.3389/fpsyt.2025.1512565

**Published:** 2025-07-14

**Authors:** Gioia Chilton, Jennifer Marie DeLucia, Myissha Tompkins

**Affiliations:** ^1^ Intrepid Spirit Center, A.T. Augusta Military Medical Center, Fort Belvoir, VA, United States; ^2^ Department of Creative Arts Therapy, College of Visual and Performing Arts, Syracuse University, Syracuse, NY, United States; ^3^ Independent Researcher, Stafford, VA, United States

**Keywords:** female service members, mild traumatic brain injury, art therapy, combat trauma, posttraumatic stress disorder (PTSD)

## Abstract

This retrospective case study explored the use of art therapy (AT) with an AfricanAmerican female combat veteran experiencing the effects of mild traumatic brain injury (mTBI) and co-occurring post-traumatic stress disorder (PTSD). The study aimed to understand how culturally informed military AT functions in the treatment of a female service member with mTBI and PTSD. Qualitative data were collected from artwork, case notes, and the therapist’s clinical reflections. Quantitative data were collected using the Emotion Regulation Strategies for Artistic Creative Activities Scale (ERS-ACA). Thematic analysis and ERS-ACA results revealed several themes. The results illustrated that art therapy supported the client to express her needs, strengths, and treatment progress; it facilitated emotional expression; supported the expression of cultural identity; and provided a method for communicating her needs to others outside of art therapy sessions. This case study underscores the potential for utilizing standardized art therapy treatment methods to benefit military-connected individuals with similar conditions.

## Introduction

The active-duty military population experiences a higher risk for traumatic brain injury due to military training and deployment. Mild traumatic brain injury (mTBI) is a serious health concern that impacts the autonomic nervous system, creating symptoms such as loss of consciousness, memory loss, and confusion (1). Longer-term functional impairments in cognitive, physical, affective, or sleep-related areas may result, affecting balance, attention, emotional regulation, and stress reactions (2–4). Post-traumatic stress disorder (PTSD) and mTBI are often co-morbid and share common symptoms, such as insomnia, fatigue, depression, and anxiety (5). Women, a growing segment of the current veteran population, report higher rates of PTSD relative to both civilian women and male veterans. Despite this prevalence, many knowledge gaps exist in the current evidence base (6–8). A systematic review of U.S. military service members who experienced mTBI revealed that men were disproportionately represented and urged further scholarship on mTBI in female service members (9), including research regarding their experiences in military healthcare (10).

Therapists receive training in cultural competency to improve the effectiveness of trauma therapy (11). Sue and colleagues (12) found key characteristics associated with effective therapy for racial/ethnic minority clients included therapists’ awareness of biases, stereotypes, and assumptions in social institutions; awareness of oneself as a cultural being capable of unintentional microaggressions (12); and awareness of the worldviews of clients. In addition to these characteristics, therapists must also consider the impact of historical traumatic experiences, such as genocide and slavery, and “ongoing individual and collective injuries due to exposure and re-exposure to race-based stress”. (13)^,p.1^ Therapists of all backgrounds are urged to develop a willingness to explore the dynamics of power and one’s own cultural prejudices, including those related to military culture (11–14).

Within military healthcare, culturally competent treatment requires awareness of how military culture impacts care delivery (14). Military service characteristics, such as branch, rank, and military occupational specialty, can affect treatment dynamics. Additionally, cultural norms may shape care uptake, including respect for authority/power hierarchy, homogeneity, and the Warrior Ethos. These norms include the tenets of honor, sacrifice, selfless service, and silent suffering (15–17). These factors are further complicated by the impact of hero stereotypes and the stigma and potential consequences associated with requesting and receiving medical services in a military system (18).

Choosing not to disclose the impact of traumas can be a matter of professional survival for service members of all genders. Cultural factors can further complicate the lack of disclosure. Remigio-Baker (19)^,p.6–7^ and team found that “non-White service members might be less likely than White service members to seek treatment to protect their professional standing and opportunities for promotion in reaction to unconscious bias, leading to worsened symptoms” particularly among those with higher educational levels. The fear of being thought of as weak due to physical and mental health disorders can result in silent suffering and long-term accrual of stress, compounding racialized trauma. When left untreated, trauma symptoms can become reinforcing and complex over time, impacting readiness. Therefore, there is a need to increase mental healthcare informed by military cultural awareness to ensure that proper treatment occurs without delay.

Historically, U.S. women veterans were excluded from combat. This historical exclusion has resulted in a lack of knowledge about how combat exposure affects women (20). Researchers call for new studies to help providers understand in depth how health conditions such as PTSD and mTBI present in women. This includes African-American women, who may have different needs, concerns, and preferences in treatment approaches (20, 21).

### Art therapy in military settings

Art therapy (AT) is a regulated health profession where patients are invited to create artwork within a psychotherapeutic relationship with a credentialed professional art therapist (14, 16). A primary assumption of AT is that creative artmaking, an activity that involves the whole brain–body, provides artistic experiences and products that reflect a person’s identity and emotional life. This allows opportunities to contemplate, integrate, and regulate emotions (22). Treatment in art therapy includes the use of concretization, metaphor, perspective taking, and reframing, which may improve symptoms of PTSD (23). Evidence shows the default mode network (DMN), the key source of mind wandering, internal thinking, spontaneous daydreams, and imaginative fantasies, is often dysregulated in service members with PTSD and TBI (24, 25). Artmaking as a creative process requires DMN thinking to operate in tandem with visual, sensorimotor, limbic, and attention networks, potentially resulting in sensory integration. This can act to regulate the autonomic nervous system (ANS) through psychophysiological attunement (22, 23). Physically, artmaking can require fine and large motor control, kinesthetic movement, and sensory processing (22). Emerging evidence indicates that AT may promote symptom reduction through improved neuroplasticity and dynamic functional connectivity between brain circuitry regions (15, 23, 26, 27).

Art therapists support patients in the creative use of art materials and in deepening their understanding of their own art processes and images using symbolism, metaphor, and artistic language to develop new self-awareness and insights, communication, and sensory integration (28, 29). AT has been used to help service members and veterans experiencing PTSD and TBI for the last 70 years (14, 16, 30, 31). Typical art therapy goals for mTBI and PTSD military service members include stress reduction, self-expression, communication, identity exploration, emotional regulation, and processing grief and trauma (15, 16) (see **Table 1**). AT may enhance effortless engagement in tasks to overcome avoidance, enable feelings of safety and relaxation, promote growth, aid in the reconsolidation of trauma narratives, improve executive functioning, and reactivate positive emotions (14, 28, 29, 34, 36, 37). AT allows for the simultaneous expression and concealment of information through symbols and metaphors, fosters agency, and leverages strengths. This provides a treatment option that may be deemed acceptable by service members, including female racial/ethnic minority service members, to address symptoms associated with mTBI and PTSD.

**Table 1:** Needs, goals, and art media variables for four art therapy interventions used in this case study and across Creative Forces sites. (15).

Art therapy intervention	Needs and goals	Art media variables (32)
Bridge with Path Drawing (33):: Service members are provided 12 × 18 drawing paper and pencils, markers, oil, and chalk pastels and asked to draw a bridge that connects to a path	• Bring to consciousness thoughts through use of symbols and/or metaphors • Explore individuals’ experiences of transitions, personal goals, future orientation, and problem-solving abilities • Assess life meaning and purpose	•2-D •Resistive (pencils) and/or more fluid (chalk pastels) •Boundary-determined (via paper size) •Moderate level of complexity and task structure •Representational
Mask making: Service members are provided with a brief orientation and paper-mâché masks and invited to alter their masks using acrylic paints, modeling clay, or additional sculptural assemblage materials (28, 34)	• Regulate emotions • Express identity/sense of self • Strengthen cohesion of intersecting identities • Improve cognition and planning • Externalization of inner states	•3-D •Resistive (inherent structure of pre-made mask) •Boundary-determined (solid composition) •Representational
Pour painting (35): Service members are instructed in a marbling technique achieved by pouring paint colors onto a canvas and then tilting to create ripples and swirls	• Communicate emotional expression • Raise emotional self-awareness • Alleviate anxiety/inhibition • Foster positive emotions • Promote mindfulness • Practice release of control	•2-D •Fluid (acrylics) •Quantity-determined (paint amount provides limit) •Moderate level of complexity and task structure •Abstract
Needle felting: Service members are instructed in a technique achieved by stabbing barbed needles into soft wool fibers, which are fused together to create fiber-art objects	• Discharge physical tension through repetitive, rhythmic motions and proprioceptive feedback • Express and regulate emotions • Practice hand–eye coordination and motor control • Sensory integration	•2-D or 3-D •Balance of malleable/resistant fibers •Quantity-determined (fiber amount and time allotted provide limit) •Low level of complexity and task structure •Abstract or representational

## Methods

### Research design

The study employs a retrospective case study design, using constructivist epistemology, thick description, and thematic analysis. The case study was constructed by a review of the first author’s clinical notes, including photographs of client artwork created during sessions, and consultation with an outside expert, the second author. The thick description was developed by author 1 using case notes, client artwork, and the therapist’s clinical reflections. Thick description is a strategy for improving the transferability of qualitative research findings by giving a thorough account of participants’ experiences. The intent is to present the case with ample detail for readers to evaluate trustworthiness (38, 39). Thick description was applied to this case study through a concise description of the sessions, artwork, client responses, and the therapist’s responses and interpretations of the sessions. Consumers of the research may then generate implications for their own practice settings or questions for future research (38).

The therapists’ reflections are presented as part of the thick description to illustrate how they attended to culturally significant aspects within the therapeutic relationship and the content of the artwork and therapy. The individual who participated in the treatment described is the third author on the manuscript. The researchers performed member checking by asking the third author to review the case study for accuracy and make any necessary changes regarding the presentation of her experiences in art therapy to ensure accurate representation of the treatment process and outcomes. The third author’s involvement in the research and dissemination of the findings aligns with current ethical best practices in research (40). As the person living with the mTBI and PTSD experience, she is the expert of her experience.

### Ethical statement

Informed consent was obtained from the service member for the use of all photographs of artwork created in art therapy clinical sessions included in this manuscript. Additionally, the service member provided informed consent and approval for the description of her treatment experience to be published in this manuscript. This study was deemed exempt from Institutional Review Board review by the Department of Research Programs of A.T. Augusta Military Medical Center.

### Participant

One client participant was selected for the retrospective case study using purposive sampling. The criteria for selection included having been treated for mTBI and PTSD with AT, feasibility of data collection (e.g., photographs of artwork were present in case notes), having completed all 12 sessions of AT treatment, and providing informed consent to participate in the study. The client selected was a 39-year-old African-American female Army veteran with three combat deployments who met all the requirements for inclusion in the study.

### Treatment setting

Outpatient concussion clinics within the U.S. Department of Defense healthcare system, known as Intrepid Spirit Centers (ISCs), provide multidisciplinary treatment to service members experiencing mTBIs (41). These clinics, part of the Defense Intrepid Network, offer art therapy. Programming aims to improve the health, well-being, and quality of life for military and veteran populations exposed to trauma, as well as their families and caregivers, by integrating creative arts therapies into patient-centered care at ISCs nationwide, including telehealth services and community arts activities. This case study focuses on an art therapy program within an ISC in the Mid-Atlantic, which addresses the complex interplay between mTBI and psychological health conditions using a data-driven, personalized, and interdisciplinary approach to improve the brain health, well-being, quality of life, and readiness of our service members and their families (41).

### Interventions

**Table 1** describes clinical art therapy interventions that are used by art therapists across the Creative Forces network. Clinical needs and goals are listed, as well as art media variables. These interventions were used in the case study presented in the subsequent thick description.

The following retrospective case study aimed to address the following research question: How does culturally informed military AT function in the treatment of a female service member with mTBI and PTSD? The case presented describes how the treatment interventions outlined above were provided in the context of culturally informed art therapy with an African-American service member dually diagnosed with PTSD and mTBI.

### Data collection

Qualitative data were collected from the therapist’s case notes, client artwork, and therapist’s clinical reflections. Quantitative data were collected using a standardized quantitative self-report measure, the Emotion Regulation Strategies for Artistic Creative Activities Scale (ERS-ACA), which was adapted for use in art therapy (42). The ERS-ACA measures how much artistic creative activity helps regulate emotions. The tool consists of 18 items (such as “When engaging in art therapy, it helps me disengage from things that are bothering me”, “makes me reflect on my emotions”, or “feel more confident in myself”), which are scored on a 1–5– Likert scale to measure participants’ opinions. The ERS-ACA has been found to have strong internal reliability, strong convergent and divergent validity, construct validity, consistency of internal reliability, and test–retest reliability (42). The ERS-ACA was completed by the client one time only, at the end of treatment.

### Data analysis

To determine how culturally informed military AT functions in the treatment of this case, author 1 developed a thick description of the case study. The thick description included the client’s presenting concerns, therapeutic interventions provided, the client’s response to the interventions, and the therapist’s interpretation of the client’s experience. In addition, the therapist’s positionality statement was included for context. The thick description was reviewed by author 3, the client, to ensure that it was an accurate account of her experience. Following this review, author 1 and author 2 conducted a thematic analysis to reveal overarching themes. Author 1 and author 2 independently analyzed the data using a three-step process: 1) highlighting key phrases (open-coding), 2) reviewing key phrases and developing initial codes, and 3) categorizing initial codes into general categories and themes. Author 1 and 2 reviewed the results of the analysis and developed final themes based on their findings. Author 3 then checked the final themes for accuracy.

The ERS-ACA was scored, and the results were included alongside the qualitative findings. Scoring consists of an overall factor and three subscale categories: avoidance strategies (which include distraction or detachment from stressful emotions), approach strategies (i.e., acceptance, reappraisal, and problem solving), and self-development strategies (which include enhanced self-identity, self-esteem, and agency).

## Trustworthiness

The four dimensions of trustworthiness were used to address the quality of the study and include credibility, transferability, dependability, and confirmability (38, 39). Credibility refers to how accurately the findings represent the experience of the participant (38). The first researcher’s extensive involvement with the case over a 12-week period promoted continuous observation and helped to support comprehensive insights about the case. In addition, member checking was used to ensure that the participant’s experience was accurately represented. Transferability is concerned with the ability of the findings to be used in other similar settings. (38) The researcher used thick description to present the case with sufficient detail so that the outcomes may be evaluated and the procedures replicated. Dependability refers to the researcher’s systematic documentation of the approach so that it may be evaluated and replicated (38). The researcher systematically documented the treatment progress through clinical case notes and photographs of the artwork. The second author served as a consultant to provide investigator triangulation by reviewing the thick description of the case, the visual representations of patient artwork, and the interpretations. Confirmability in case study research involves researchers being aware of how their values and biases may impact the study and presentation of the case (39). The research addressed confirmability by engaging in reflexive practice and including a positionality statement in the presentations of this case (43).

## Retrospective case study: thick description

### Art therapist positionality statement

I (first author) present my background to provide context for the role of the art therapist in the following case study. I am a 56-year-old heterosexual, non-disabled, U.S.-born, White, civilian woman. I have practiced as an art therapist since 1994 and with military service members since 2019. I was raised in Miami, Florida, have financial and social resources, and am the youngest child with learning disabilities. I later attended graduate schools in the Mid-Atlantic area. My heritage includes Scottish and English ancestors who immigrated to North America circa 1700s–1800s. I am most aware of my roles as a spouse, mother, art therapist/art therapy scholar, friend, and artist.

### Early stage of treatment

At the time of intervention, Myissha was a 39-year-old Sergeant, First Class, African-American female Army veteran with three combat deployments recovering from PTSD. She was a contract specialist, an administrator in charge of military procurement systems, and had two sons who were 19 and 6 years old. Her worst TBI was during her first deployment in Iraq in 2004 when she fell from a truck while unloading supplies, hitting her head on concrete. In another incident, she was trapped between two suicide bombers when her convoy was hit. She was referred for AT from her medical provider after reporting excruciating headaches that had worsened in 2019, as well as sleep disturbances, anger, anxiety, depression, grief, and loss. She was also in the process of retiring from the Army. She was engaged in physical therapy for knee and cervical pain; group music therapy for coping and stress reduction; speech language pathology to address communication, attention, and memory concerns; and behavioral health sessions to address anxiety and adjustment issues.

After an initial art therapy assessment in April 2021, Myissha engaged in 12 one-hour AT sessions from November 2021 to March 2022. Individual sessions were held in person in a well-stocked art therapy studio located in the clinic. The initial task for treatment was to set internally motivated goals. To facilitate this task, I asked Myissha to complete a Creative Forces standardized art therapy intervention called Bridge Drawing with Path (BwP) (see **Table 1**). BwP is an art-based assessment designed to assess life transitions, perceptions of environment, possible conflicts or barriers, problem-solving abilities, and future orientation (33). Participants are offered 12 × 18-inch white drawing paper and drawing materials and are directed as follows: “Draw a bridge from someplace to someplace. The bridge connects to a path. Draw the path and write where the path leads you to”. Upon completing the drawing, I asked Myissha open-ended questions regarding the meaning of the image and how it related to her life goals.

**Figure 1:**
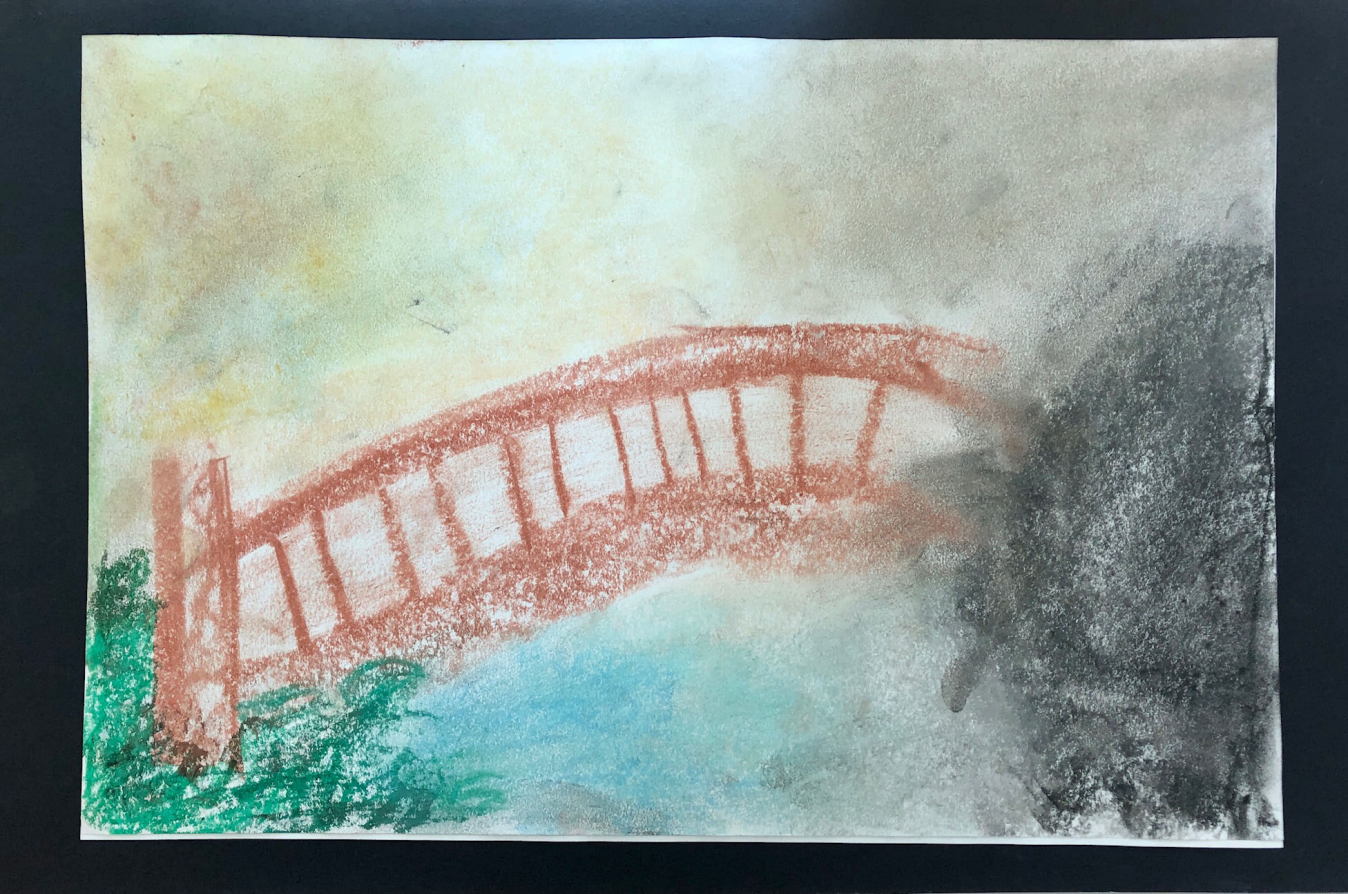
Myissha Tompkins, Bridge Drawing with Path titled, “To Overcome”. Used with permission.

Myissha described her bridge as transitioning from the “innocence” of the past (**Figure 1**), where she felt a “sense of confidence” (left), to the “darkness” of war (right). She stated, “I wish it was in reverse”, referring to her desire to have it progress from darkness to the brighter area. “I have to live with the darkness”, as “there are triggers all around”, describing her need to “suppress it [triggers], to stay above ground”. She described, “It’s dysfunctional, but it’s pretty, all of it”. When identifying future goals, she said, “It’s not too late to have brighter days, but right now I’m stuck”. The completed artwork visually depicted how the past influenced her present, yet also indicated hope as she described, “It’s not too late”. She titled the image “To Overcome”, which indicated a sense of hope and possible movement toward acceptance. She stated that her children were a motivating factor in her desire to overcome the darkness.

### Middle stage of treatment

Over the next several months, Myissha created a series of mixed media artwork and paintings. She was offered mask-making molds (see **Table 1**) and began by gluing patterned, colorful tissue paper scraps onto the surface of a brain-shaped mold to create a sculpture. Mask making was offered as an intervention to support self-expression and strengthen the cohesion of intersecting identities. While working, she discussed various topics such as parenting her youngest child; the mix of stress, worry, and pride regarding her older son entering the military; the challenge of buying her first home; the impact of the military-to-civilian transition on her sense of identity; and how her faith sustained her though these challenges. As the art therapist, I provided active listening, validation and reframing, social support, psychoeducation, and artistic technical support. Myissha reported that when she showed her social worker her art, it helped them to communicate better about her concerns.

**Figure 2:**
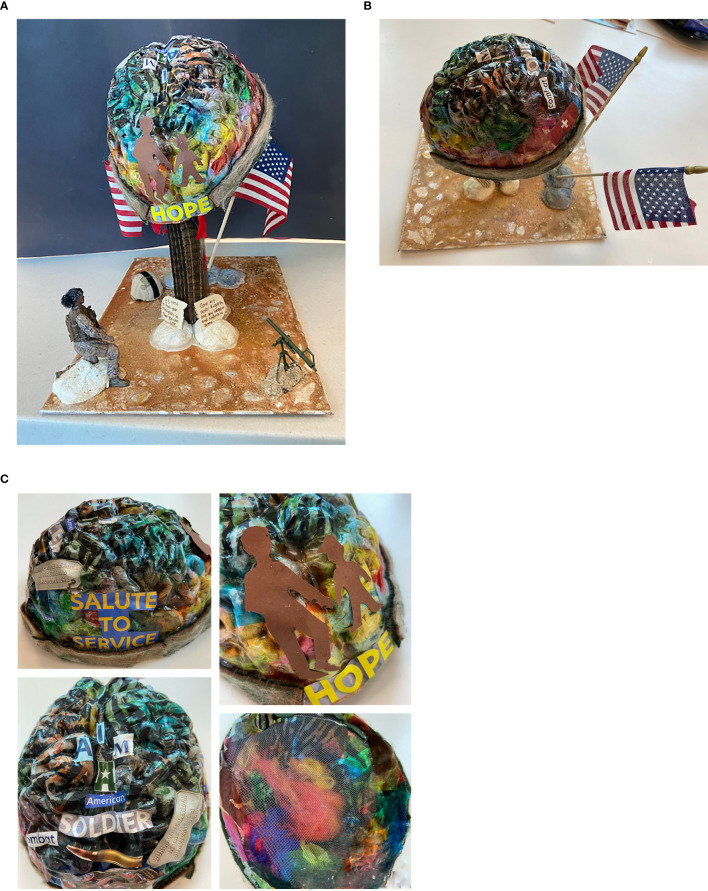
**(a, b)** Myissha Tompkins’ mixed media sculpture. At the base of the sculpture, the plaque reads, “brothers and sisters in arms” and “it’s time to free your thoughts so you can live”. Used with permission. **(c)** Details of sculpture, including interior view showing fibers used to symbolize “fuzzy thoughts” of brain fog. Used with permission.

Over the next few sessions, Myissha continued to work on her brain sculpture, gluing on magazine collage words such as “hope” on one side and “I am an American Soldier”, “combat”, “Afghanistan”, and “salute our service” across the other sides of the surface (see **Figures 2a, b**). During one session, she shared that she was feeling more anxious “for no reason” over the last few days. I provided a 15-minute guided “safe place” meditation and visualization exercise to induce feelings of security, after which she stated she felt calmer. She was excited at times about her sculpture as it developed and shared what she thought about its design in between our sessions. I recognized that Myissha was engaging in DMN thinking—creative daydreaming, consolidating her memories, and experiencing moments of self-reflection that could help integrate trauma narratives.

To assist Myissha with grounding or reconnecting with the present moment when feeling anxious or overwhelmed, I introduced her to needle felting (see **Table 1**). This technique involves the kinesthetic action of punching fibers into cloth using a needle tool. The repetitive motions involved in needle felting can help to release physical tension and foster sensory integration. Myissha engaged in needle felting after selecting wool roving in colors that reminded her of her uniforms during different deployments, such as tan, brown, and green. She felted these by stabbing the needle tool into the soft wool fibers to secure them to a stable base, creating a textured fabric. During the process, she shed tears, recalling the loss of her innocence during her first deployment at age 21. I offered compassion and encouragement as she expressed herself and spoke of reconnection to her faith. My aim was to model acceptance of emotional experiences, to aid in the co-regulation of emotions, and to support her in accessing her strength of spirituality.

### Late stages of treatment

As treatment progressed, Myissha reflected on her treatment, reporting that she did not feel hurried or rushed in art therapy. She also reported feeling a sense of acceptance regarding life’s challenges. Recognizing that her sense of safety had increased, I offered 3-D, sensory-rich art materials to safely activate the brain’s somatosensory processing system and to regulate sensory activation. Over time, she worked on her brain sculpture and removed some “fuzzy thoughts” (created from wool roving) from the brain area and constructed a “screen” to hold in some thoughts and let others go (see **Figure 2c**). She added the needle-felted fabric with hot glue to strengthen the edges of her brain, which began to look like a helmet. She used a stencil to draw figures to represent her sons, which she cut out and glued on the brain sculpture to illustrate how they are always on her mind. On a cognitive level, Myissha’s evolving artwork offered powerful symbolic expression about that which was most important to her.

During the next several sessions, Myissha and I took a break from constructing her brain sculpture to try pour painting, a standardized Creative Forces’ art therapy intervention (15) (see **Table 1**), a marbling technique achieved by diluting paint and simply pouring it onto a surface. The poured paint then forms delightfully intricate ripples and swirls and fosters spontaneity, present-moment awareness, and positive emotions such as wonder. The break from the brain sculpture was initiated to provide some psychological distancing, time for reflection, and an opportunity to utilize coping skills before returning to complete the project. For her pour painting, Myissha chose metallic and earth tones and seemed to enjoy the process of mixing and stirring colors, adding textural details using sand and glitter to create a taupe and beige landscape. She was pleased with the final product and spoke about how she may have an art studio in her new home to use art making for stress management.

The following week, she felt low in mood. She reported that she had a busy holiday visiting her sister and seeing her sons. She had awoken last night in a startle response, jumping up out of bed feeling agitated and fearful, but with no memory of a nightmare. Although tired, she created a new self-directed painting titled “The places life will take you …” using textural collage elements, while sharing hopes and dreams for 2022, remarking, “this is MY year”. She shared plans for her retirement ceremony and post-retirement plans, which included giving back to veterans through service work. I observed over time how her creative endeavors reinforced her role as an artist and may have boosted her mood and optimism for the future.

The next session, Myissha arrived sharing about her pain in the back and hip areas, “that’s just how it is sometimes,” she said, indicating acceptance. We discussed pain as a brain–body experience and how stress management techniques could be used to address chronic pain. Psychoeducation regarding pain was offered to help normalize her experience and provide information on the recovery process. She attached the brain/helmet to a post anchored into the solid base of the sandy landscape she had created the previous session (see **Figure 2a**) with hot glue. She cut out red fabric to drape below the brain, creating the appearance of dripping blood, thus viscerally expressing the experience of TBI. She remained emotionally regulated throughout this part of the sculpture’s construction. In the next session, she added flags and attached the post to the base. With air-dry clay, she molded rocks and colored them in tan and brown colors while mentioning how the artwork could be like a map. As she worked, she named the places in Afghanistan and Iraq where she had deployed. I attributed her ability to discuss the locations of her deployments without it triggering symptoms of PTSD to her growth over time and our well-established therapeutic alliance. This seemed a valuable moment of empowerment in which she reclaimed and made meaning of her experiences, inscribing on a small wood piece, “Gone but never forgotten are my brothers and sisters in arms/it’s time to free your thoughts so you can live”. Finally, she used the hot glue gun, this time with a tremor in her hand, and added the broken wooden tablet, like a gravestone, to the base of the sculpture.

**Figure 3:**
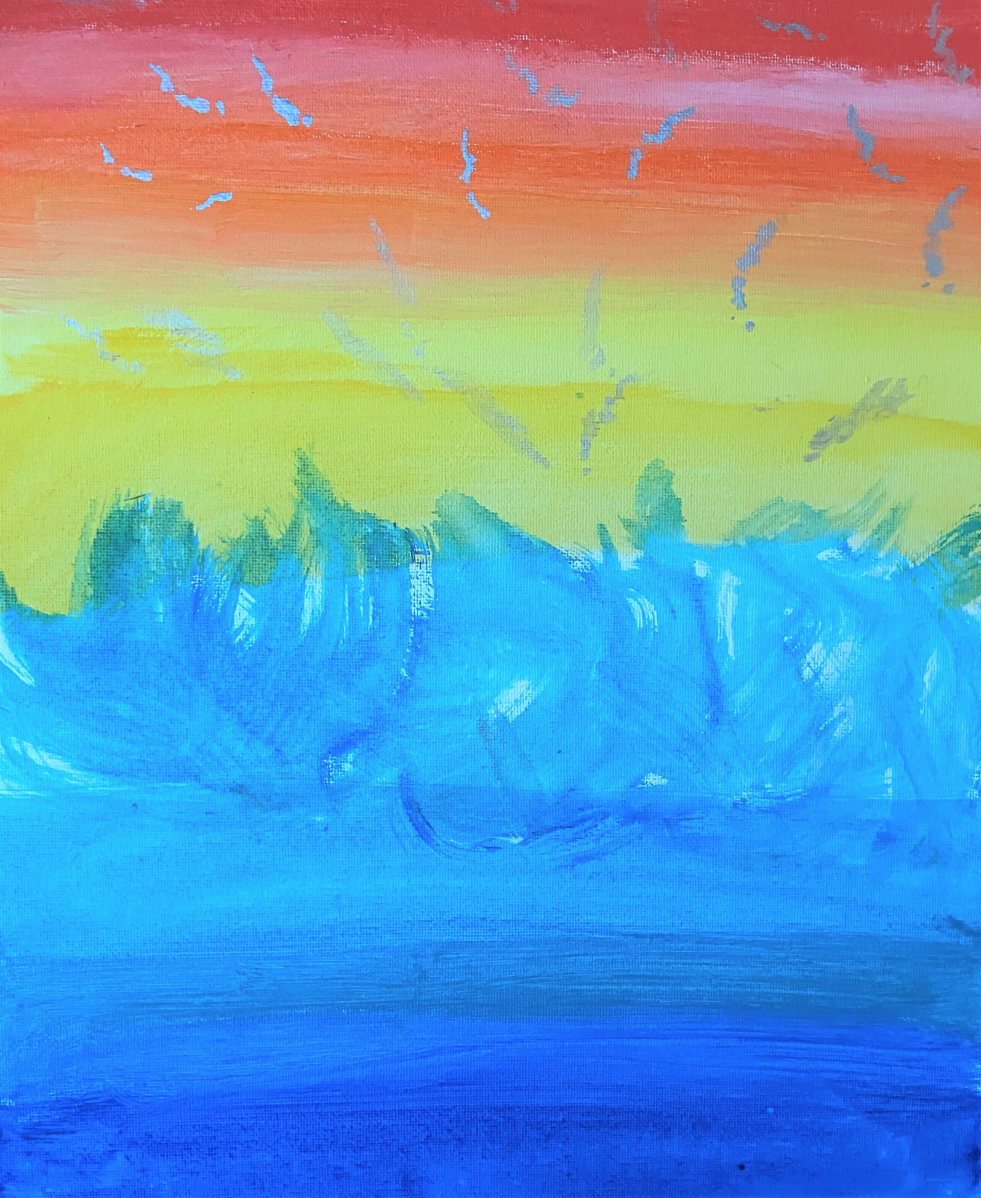
Myissha Tompkins, “Calm”, acrylics on canvas board painting. Used with permission.

In the next session, Myissha chose not to work on the sculpture. She stated she felt calm this day and wanted to enjoy her positive mood, so she chose instead to paint. The powerful work of the prior session may have needed to be integrated further; fortunately, art making can promote play and enjoyment, as well as the expression of grief. By supporting Myissha’s need to self-direct in session, I reinforced her ability to attend to her emotional needs. She used blue colors and was experimental in her use of brushes. She finished a self-directed painting of a playful blue figure and then chose to paint on another canvas soft ombre shading, colors which she said reminded her of the beach (see **Figure 3**). She was provided with additional art materials to take with her to begin her home art practice, to support her independence in her role and practice as an artist.

Toward the end of treatment, Myissha focused on overcoming the technical challenges of the sculpture (**Figure 2a**), such as how to attach items. Using multiple kinds of glue, she securely attached the brain/helmet to the base. I offered her a box of various found objects, including soldier figurines. She added “hair” (wool roving) to the African-American soldier figure to transform it into a representation of a female soldier. She discussed the importance of her connection to her fellow service members, stating that she someday wanted to be buried in Arlington Cemetery to be with them. While discussing her sculpture, Myissha said, “I want it to tell my story—where I go in my head sometimes—constantly going back to ‘04—and I want my art to speak for my colleagues as well”. She took the completed sculpture with her to display at home. As she completed treatment, we reviewed many possible strategies for lifting mood, such as practicing self-compassion, spending time outdoors, gratitude journaling, aromatherapy, communicating with her youngest son’s school, preparing to use VA behavioral healthcare, artmaking, and the use of the DHA PTSD Coach app (44). Myissha was also provided with information regarding local community arts engagement classes, which help veterans build resilient communities and adapt to civilian life.

### Art therapist’s clinical reflections

Receiving social support, artistic support, validation, and positive attention through art therapy seemed useful for Myissha. Together, we were able to create a therapeutic alliance across our different life experiences, forming a positive attachment that withstood the unavoidable moments of relational rupture. I knew that as a White civilian woman, I could never totally understand her experience as a Black female combat veteran. My goal was, nevertheless, to create a psychological, physical, and artistically safe space for her to create and be witnessed. The BwP drawing (**Figure 1**) was useful, as it helped us both to see visual evidence of her traumatic past and celebrate her sense of hope and acceptance of the whole of her military experience. As Myissha transitioned from active duty to veteran, she processed loss and grief through working on her sculpture (**Figure 2**) and at times paused trauma processing to create joyful art (see **Figure 3**).

I witnessed as she wondered if she had made the correct decision to retire, noting that as her work pace slowed down pre-retirement, she had time to reflect on the impact of recent changes. Military-to-civilian transitions can be a stressful time, as it is a period when not just one’s career but also one’s entire personal identity may be in flux. Reflecting on this process, I hoped Myissha found it useful to have moments to contemplate and process her feelings. Knowing the importance of faith through what she communicated verbally and showed in her art, I aimed to reinforce faith as a protective factor. Many times, Myissha referenced her faith, and I tried to affirm her belief in her higher power and her efforts to seek support through her church community.

A moment that stood out for me was when Myissha selected the Black soldier figure and added a bit of black wool roving to create the figure’s hair in a low bun, which seemed to instantly transform the figure into a Black female soldier. This moment felt like a victory, as Myissha used her creativity to overcome the lack of Black female figures. There was a moment when, together, we acknowledged that, while not the typical hero stereotype, Black female soldiers had indeed fought in the recent wars. They were not invisible; their service mattered! The power of Myissha’s art to communicate this through metaphor was striking.

As Pollman-Turner (45) writes, “If, like me, you’re a white provider working with a minority service member, race is already in the room. Directly inviting your patients to share their racial experiences and perspectives signals that therapy is a space where the service member can be more fully authentic, ‘heard’, and understood, which can be an incredibly powerful and healing experience”. Myissha’s healing experience included attaching the tiny bundle of wool fibers to represent hair—a topic laden with meaning, particularly for African-American female service members who must conform to military grooming standards (46, 47). The artwork she created within the supportive setting thus expressed her cultural identity and what she found important, such as the “hope” she found in viewing her sons as illustrated in her artwork (**Figure 2c**).

As treatment ended, Myissha anecdotally shared that art therapy had been helpful, specifically in helping her to better communicate with her social worker, providing stress relief, processing trauma, and making meaning of her combat experiences, as well as finding areas of post-traumatic growth, such as developing her identity as an artist. In terms of goals, art therapy seemed useful in addressing grief and loss, emotional regulation, sensory integration, and her sense of self/identity development during a time of military-to-civilian transition. At discharge, Myissha expressed her opinion that art therapy would have been helpful earlier in her military career. She was interested in sharing the benefits of art therapy with other veterans and service members, which was one of her motivations for serving as a co-author on this manuscript. She described a need for military healthcare to increase access to treatment options that build resiliency, which mTBI and PTSD patients of all genders and varied cultural backgrounds may find beneficial.

## Case study results and discussion

Thematic analysis and review of the self-report measure and client artwork revealed several themes (see **Table 2**). The results illustrated that art therapy supported the client to express her needs, strengths, and treatment progress; it facilitated emotional expression, supported the expression of cultural identity, and provided a method for communicating her needs to others outside of art therapy sessions.

**Table 2:** Themes of how art therapy functioned during the treatment of an African-American female combat veteran with mTBI and PTSD across early, middle, and late stages of treatment.

Theme	Early	Middle	Late
Art therapy supported expression of needs, strengths, and treatment progress	“I wish it was in reverse”, referring to her desire to have it progress from darkness to the brighter area “It’s not too late to have brighter days, but right now I am stuck.”	Shared she was feeling more anxious “for no reason” Discussed various topics such as parenting her youngest child and the mix of stress, worry, and pride regarding her older son entering the military, etc. Reported that when she showed her social worker her art, it helped them to communicate better about her concerns	Reported feeling a sense of surrender or acceptance regarding life’s challenges Created a new painting titled, “The places life will take you …”
Art therapy supported emotional expression	“I have had to live with the darkness” as “there are triggers all around” “It’s dysfunctional, but it’s pretty, all of it.”	She shed tears, recalling the loss of her innocence during her first deployment at age 21 Continued to work on her brain sculpture, gluing on magazine collage words such as “hope” on one side	[She] added the broken wooden tablet on the base of the sculpture, which stated, “Gone but never forgotten are my brothers and sisters in arms/it’s time to free your thoughts so you can live” While sharing hopes and dreams for 2022, remarking, “this is MY year.”
Art therapy supported expression of cultural identity		Continued to work on her brain sculpture, gluing on magazine collage words such as “I am an American Soldier”, “combat”, “Afghanistan”, and “salute our service” Engaged in needle felting after selecting wool roving in colors, which reminded her of her uniforms during different deployments, such as tan, brown, and green. [She shared] how her faith sustained her through these challenges	She added “hair” (wool roving) to the African-American soldier figure to transform it into a representation of a female soldier. Used a stencil to draw figures to represent her sons, which she cut out and glued on the brain sculpture to illustrate how they are always on her mind. Discussing her sculpture, Myissha said, “I want it to tell my story—where I go in my head sometimes—constantly going back to ‘04—and I want my art to speak for my colleagues as well.”

mTBI, mild traumatic brain injury; PTSD, post-traumatic stress disorder.

The ERS-ACA scores indicated the art therapy experience was highly useful for emotion regulation (general factor score, 4.38); on the three sub-factors, Myissha scored the emotional regulation strategy of avoidance highest (4.85), i.e., engaging in artmaking was found most helpful by distracting attention from negative emotions. She also highly endorsed approach strategies (4.33) and self-development strategies (4.0).

### Art therapy supported expression of needs, strengths, and treatment progress

Art therapy supported the expression of psychological needs, strengths, and psychological growth. One example from Myissha’s artwork was the removal of “fuzzy thoughts”, which may represent her recovery from the brain fog associated with mTBI, and the ability to take control over potentially negative thoughts and refocus on the positive. She demonstrated the ability to self-regulate during times when she chose to pause on her brain sculpture and work on a new project, like she did with pour painting. Later in treatment, she stated that in art therapy sessions, she did not feel rushed. This aligns with one of the benefits of art therapy; it is known for allowing clients to process emotions at a manageable pace (48). The artwork produced during sessions also helped Myissha communicate her needs and progress to others. For example, she discussed sharing her artwork with her social worker to foster communication. Sharing artwork as a form of communication can provide a holistic way to reflect a person’s subjective experience while also highlighting his/her specific cultural and social contexts (49).

### Art therapy supported emotional expression

Art therapy supported the expression of a range of emotions, including anxiety, enjoyment, hope, loss, and acceptance. Evidence is seen in multiple moments of visual and verbal self-expression of feelings recorded in the thick description and the high score marked on the ERS-ACA scale, indicating that artistic creative activity helped to regulate emotions (general factor score, 4.38). As discussed, AT can promote expression and concealment simultaneously through the use of symbols and metaphors (35, 50). The flexible nature of the process can support participants in driving the pace of therapy, promoting a sense of psychological safety (51, 52). Different cultural norms associated with emotional expression can be honored as the client remains in control over the process (11, 49, 50).

### Art therapy supported expression of cultural identity

Art therapy supported the expression of cultural identity. Myissha’s artwork reflected on many facets of her identity, including her military identity as a female African-American soldier, a mother, a woman of faith, and a person experiencing symptoms of mTBI and PTSD. Myissha used the art materials provided to express these various aspects of her identity. In one session, she added hair on a soldier figurine she placed in her sculpture and shared that she wanted her art to tell her story and speak for fellow soldiers. Exploring the many intersecting aspects of Myissha’s identity was an important part of the treatment. Her family and faith were grounding and served as a source of inspiration for her to focus on treatment. This is significant, as social support and religion have been identified as important protective factors that can mitigate risk for suicide in African-American women veterans (53).

## Limitations

The case study presented may have issues with internal validity as the study took place in a naturalistic treatment setting, as is common with case study research, and, therefore, the researcher was not able to limit the impact of other variables on treatment outcomes. The client was involved in several other treatments during the time she was participating in art therapy. In addition, there was potential confirmation bias in the selection of the case. We recommend that future case studies use clear selection criteria with random sampling or employ a comparative case study design to highlight variations in successful and unsuccessful outcomes (48).

## Conclusion

Section 7.2 of the Ethical Principles for Art Therapists states art therapists should “learn about the belief systems of people in any given cultural group in order to provide culturally relevant interventions and treatment”. In a recent position paper, the American Art Therapy Association Ethics Committee (54)^,p.1^ urged, “It is essential that art therapists seek culturally sensitive education and supervision on issues around race and racial trauma so that we reduce the risk of re-traumatization and do not burden our clients of color to become our teachers in areas involving racial trauma”. However, clients often do become the primary teachers for their therapists regarding the nature of their unique experience. Current ethical best practices in research align with the motto, *nothing about us, without us (* 40 *).* The current study gains relevance and credibility by engaging a consumer of healthcare as a health educator through their participation in this scholarship.

This case study illustrated how AT embedded within a military healthcare system functioned with an African-American female military service member experiencing mTBI and PTSD. While initial evidence suggests that art therapy benefits military populations experiencing mTBI and/or PTSD through improved dynamic functional connectivity between brain circuitry regions (23), more robust studies, which include women and consider cultural factors, are needed to explore mechanisms of change, dose, and treatment fidelity issues (29, 34, 37). The areas of future inquiry could include change factors related to the therapeutic relationship in art therapy, possible differences in outcomes in intensive vs. longitudinal models of outpatient art therapy, and whether specific art therapy interventions are more or less effective for female service members recovering from PTSD and mTBI in the short and long term (34, 37).

Military cultural considerations, such as the Warrior Ethos, as well as ethnic and cultural factors that intersect with gender, age, and sexual orientation, must also be addressed in treatment (17). AT may be useful to circumvent the military cultural norm of silent suffering by providing opportunities for visual expression of personal cultural identity, and contemplative and spiritual meaning-making (15). As illustrated in this case study, art therapy may provide an outlet for exploring intersecting identities as participants craft unique representations of their internal experiences, like Myissha did with her brain sculpture that included references to her many roles, such as a mother, as a grieving Army soldier, and as a person recovering from trauma.

This case study underscores the potential for utilizing standardized art therapy treatment methods, like those used in this case study (see **Table 1**), to benefit military-connected individuals with similar conditions. Additionally, it demonstrates the need for art therapists to practice reflexivity, encouraging clients to “bring their whole self into the therapeutic space”, (11) ^(p. 65)^ and working together with clients to express and explore their experiences.

## Author’s note

The opinions contained herein represent the private views of the authors and are not to be construed as official or as reflecting the views, opinions, or policies of the Department of Defense or the U.S. Government. Mention of trade names, commercial products, or organizations does not imply endorsement by the U.S. Government. The identification of specific products, scientific instrumentation, or organizations is considered an integral part of the scientific endeavor and does not constitute endorsement or implied endorsement on the part of the author, DoD, or any component agency. This material was created free of branding or market affiliations. The authors are operating solely as contributors.

## Data Availability

The original contributions presented in the study are included in the article/supplementary material. Further inquiries can be directed to the corresponding author.
